# Atrioventricular node ablation and the pathological findings of a refractory ectopic atrial tachycardia in a small infant with hypoplastic left heart syndrome: a case report

**DOI:** 10.1093/ehjcr/ytae493

**Published:** 2024-09-10

**Authors:** Masayoshi Mori, Chihiro Ichikawa, Taka-aki Matsuyama, Risa Nawa-Hasegawa, Hisaaki Aoki

**Affiliations:** Department of Pediatric Cardiology, Osaka Women’s and Children’s Hospital, 840 Murodo-cho, Izumi, Osaka 594-1101, Japan; Department of Pathology, Osaka Women’s and Children’s Hospital, 840 Murodo-cho, Izumi, Osaka 594-1101, Japan; Department of Legal Medicine, Showa University School of Medicine, 1-5-8 Hatanodai, Shinagawa, Tokyo 142-8555, Japan; Department of Pathology, Osaka Women’s and Children’s Hospital, 840 Murodo-cho, Izumi, Osaka 594-1101, Japan; Department of Pediatric Cardiology, Osaka Women’s and Children’s Hospital, 840 Murodo-cho, Izumi, Osaka 594-1101, Japan

**Keywords:** Atrioventricular node ablation, Hypoplastic left heart syndrome, Atrial tachycardia, Histological evaluation, Paediatric, Case report

## Abstract

**Background:**

An atrioventricular node (AVN) ablation and permanent pacing have been previously reported as effective treatments for patients with atrial tachyarrhythmias. However single-ventricle patients requiring chronic ventricular pacing are at a higher risk of developing ventricular dysfunction and atrioventricular valve regurgitation. We report a case of successful AVN ablation in a 3-month-old infant with hypoplastic left heart syndrome and ectopic atrial tachycardia (EAT).

**Case summary:**

A boy with hypoplastic left heart syndrome who had a refractory EAT resistant to various medications. At 2 months old, we performed an urgent radiofrequency (RF) catheter ablation of the EAT and the applications delivered at the cavo-atrial junction. Although it disappeared after the first catheter ablation for 2 weeks, it recurred on the next day after the diaphragm plication. At 3 months old and weighed 3.1 kg, we decided to perform an urgent AVN ablation of the EAT. The application was performed on the mid-septum of the tricuspid septum. A permanent pacemaker was implanted after the ablation. After the AVN ablation, the haemodynamics stabilized during the EAT. However, he died from a bacteraemia infection at 4 months.

**Discussion:**

This patient received an AVN ablation due to failure to previous RF catheter ablation and was haemodynamically stable with the dual-chamber pacemaker. The AV block was successfully created by RF energy on the mid-septum of the tricuspid annulus in this hypoplastic left heart syndrome patient. Pathological findings exhibited that the compact AVN was totally ablated without damage to the tricuspid leaflets or coronary artery.

**Learning points**

- Atrioventricular (AV) node ablation and pacemaker implantation were effective in small infant with a life-threatening atrial tachycardia.
- Atrioventricular node ablation was achieved on the mid-septum of the tricuspid septum in hypoplastic left heart syndrome.
- Pathological findings proved that the compact AV node was completely cauterized without damage to the tricuspid leaflets or coronary artery.

## Introduction

Ablation of the atrioventricular node (AVN) and implantation of a VVI pacemaker can control the ventricular rate in patients with atrial tachyarrhythmias and heart failure when medication or catheter ablation fails to control the rate and disease symptoms. Atrioventricular node ablation is a class IIb indication for patients with atrial tachycardia and congenital heart disease based on the PACES/HRS expert consensus statement.^1^ Even in Fontan patients who are drug resistant or do not respond to catheter ablation, AVN ablation with pacemaker implantation is an effective therapeutic option. In young children weighing <15 kg, AVN ablation is also an IIb indication when accompanied with atrial tachyarrhythmias refractory to all medications and for substrate-targeted catheter ablation who are not candidates for surgical therapy.^1^ A His bundle ablation and permanent pacing have been previously reported as effective treatments for infants with congenital junctional ectopic tachycardia.^2,3^ However, small children have been shown to be at an increased risk of complications during catheter ablation such as death, heart block, cardiac perforations and effusions, and coronary injury.^2^ Single-ventricle patients requiring chronic ventricular pacing are at higher risk of developing ventricular dysfunction and atrioventricular valve regurgitation.^4^ We report the case of a 3-month-old boy with hypoplastic left heart syndrome who had refractory ectopic atrial tachycardia (EAT) that was resistant to various medications, catheter ablation, AVN ablation, and pacemaker implantation. A pathological evaluation of the ablation lesions was performed.

## Summary figure

**Figure:**
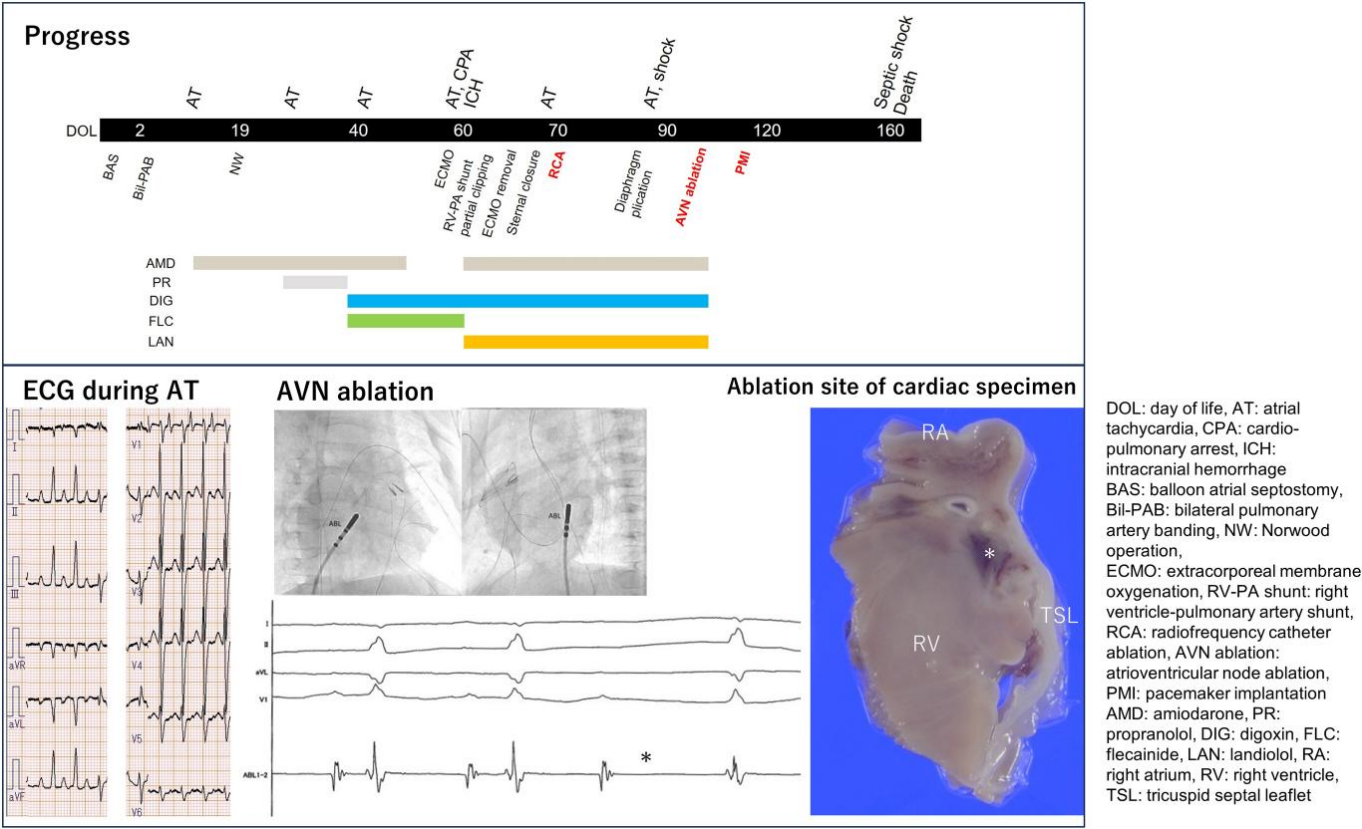


## Case presentation

A male infant, prenatally diagnosed with hypoplastic left heart syndrome, was born at 38 weeks of gestation. Postnatal echocardiography confirmed a hypoplastic left heart syndrome with mitral and aortic atresia.

**Figure 1:**
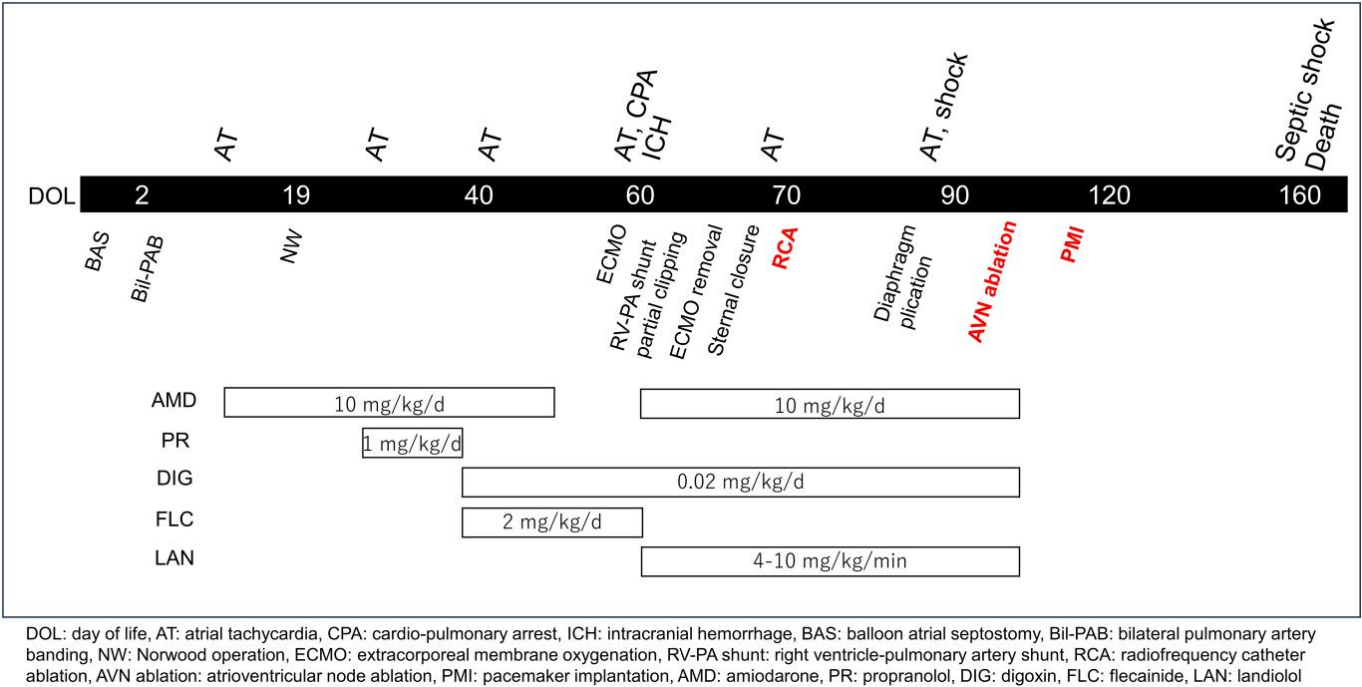
The timeline of the sequential events.

**Figure 2:**
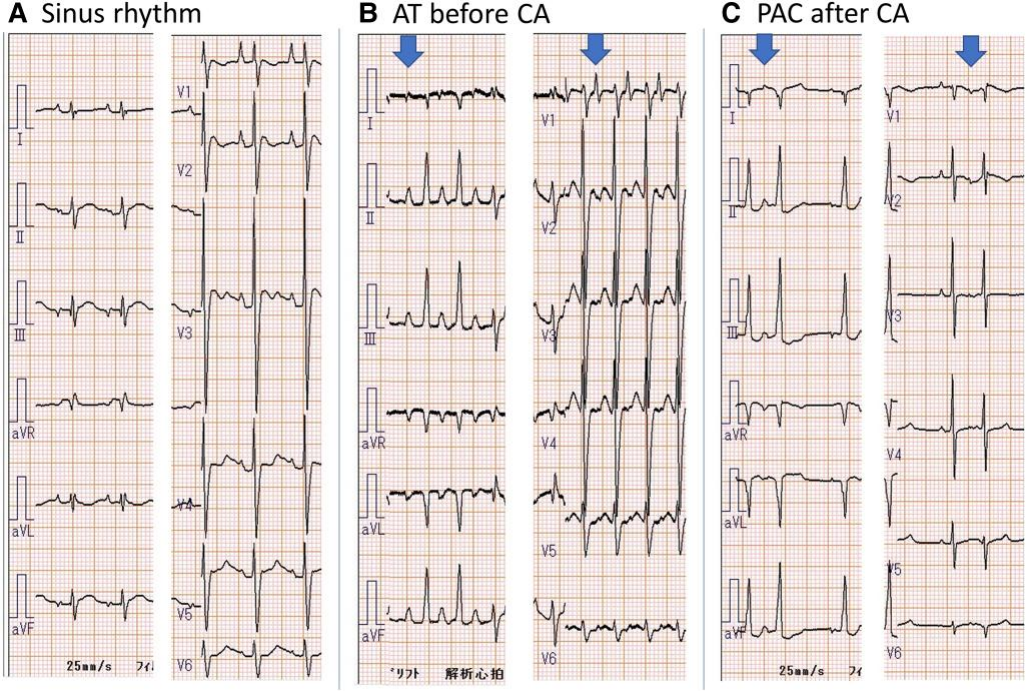
(*A*) Twelve-lead electrocardiogram during sinus rhythm. The shape of the P-wave was positive in lead I, negative in aVF, and positive and sharp in V1. (*B*) Twelve-lead electrocardiogram during atrial tachycardia before ablation. The arrows indicate the position of the P-wave. The patient’s heart rate was 220 b.p.m. The shape of the P-wave is positive–negative–positive in I, positive–positive in aVF, and positive–negative in V1, which is different from the shape of the P-wave in the sinus rhythm. (*C*) Twelve-lead electrocardiogram during atrial extrasystole seen after ablation for atrial tachycardia. The arrows indicate the position of the P-wave. The shape of the P-wave was positive in I, aVF, and V1. The P-wave differed from the P-wave during atrial tachycardia prior to ablation.

**Figure 3:**
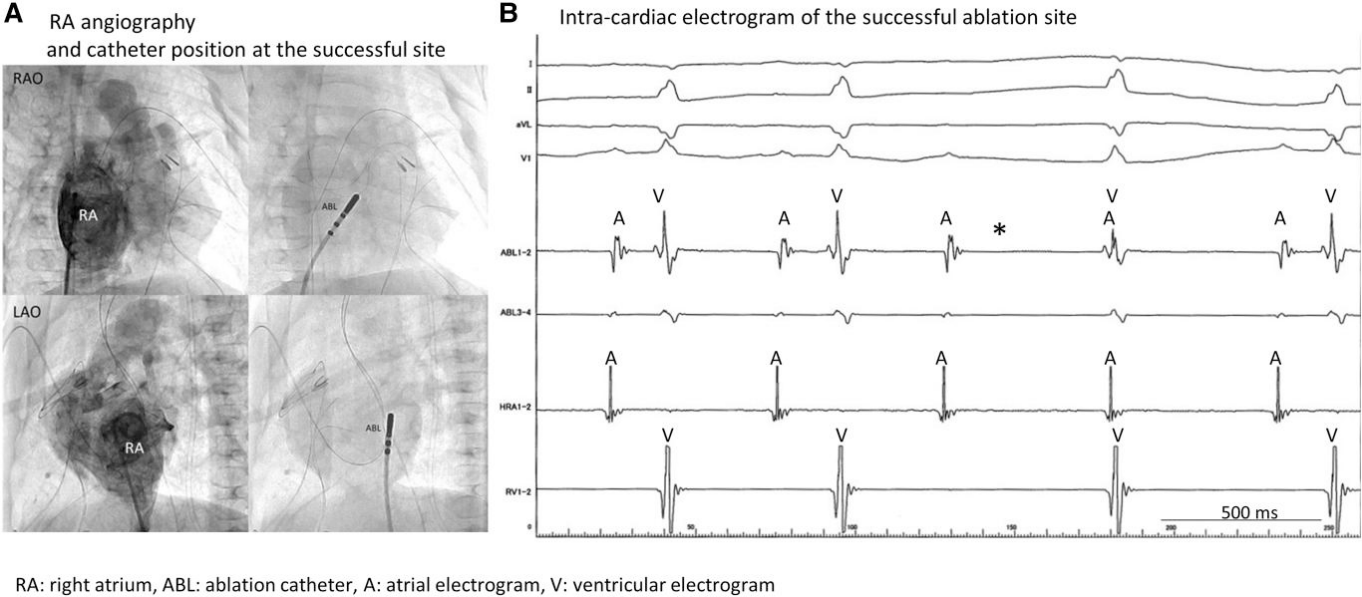
(*A*) Right atrium angiography and catheter position of the successful ablation site. (*B*) Intra-cardiac electrogram of the successful ablation site. Complete atrioventricular block was achieved in the anatomic atrioventricular node when ablation catheter contact induced an atrioventricular node block. The potentials of each patient were not recorded.

**Figure 4:**
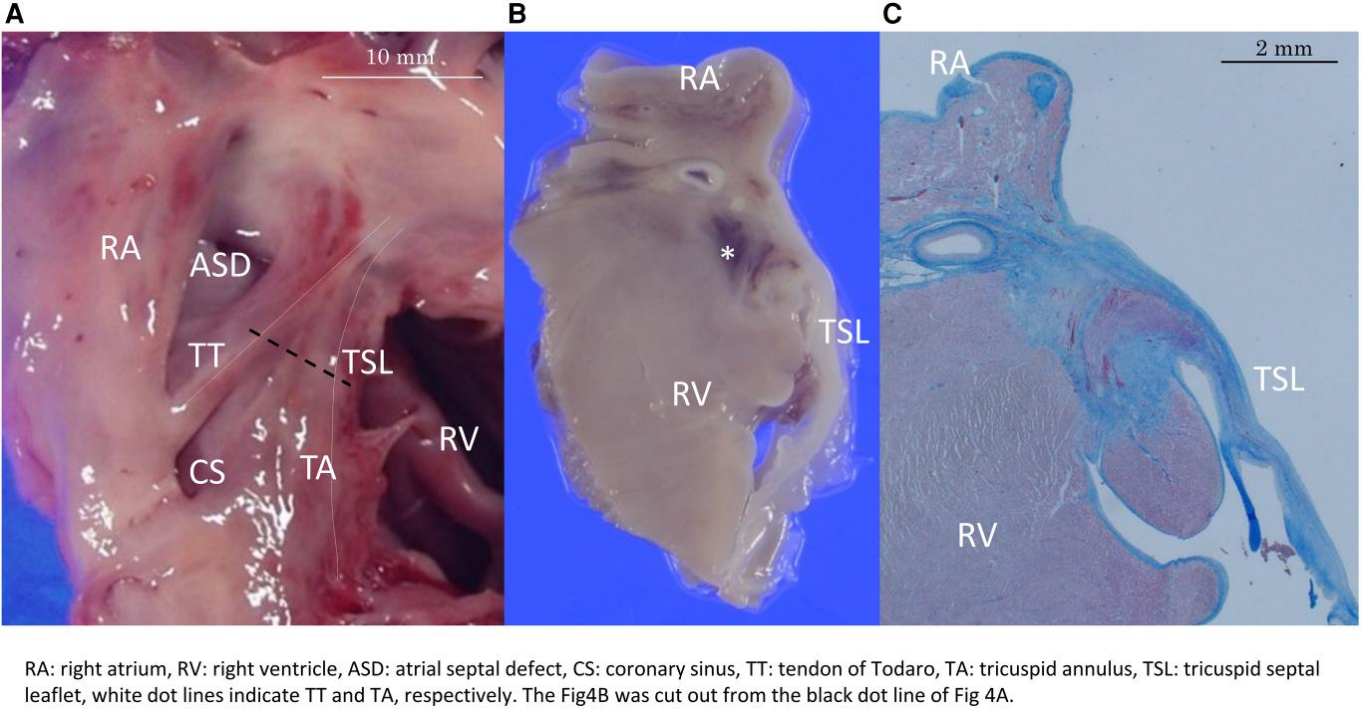
Pathological findings after the atrioventricular node ablation. (*A*) Macroscopic view of the Koch triangle. (*B*,*C*) Necrotic ventricular muscle, haemorrhage, and fibrosis replacing most atrioventricular node were observed at locations corresponding to the ablation site. The fibrosis lesion was 2.8 mm × 1.9 mm; no thromboses were observed. The tricuspid valve exhibited no significant injury due to catheter ablation.

*Figure 1* shows the timeline of sequential events. He underwent balloon atrial septostomy at 1 day of life (DOL) and bilateral pulmonary artery banding at 2 DOL. On the 12th DOL, he experienced an EAT episode (heart rate, 220 b.p.m.; *Figure 2B*), which was controlled using continuous intravenous administration of amiodarone (10 mg/kg/day). At 19 DOL, he further underwent Norwood’s procedure using a 4 mm shunt from the right ventricle (RV) to the main pulmonary artery and atrial septectomy. The AT was managed using amiodarone and propranolol. Three days after the extubation (40 DOL), he had recurrent EAT episodes, which were managed with three-combination antiarrhythmic therapy with amiodarone, digoxin (0.02 mg/kg/day), and flecainide (2.0 mg/kg/day). At 2 months of age, cardiac arrest was triggered by the EAT. Extracorporeal membrane oxygenation had also been established. Brain computed tomography revealed an acute large middle cerebral artery infarction in the right region. After extracorporeal membrane oxygenation, the EAT disappeared, and the extracorporeal membrane oxygen was decannulated 5 days later. Partial shunt clipping was performed to reduce the atrial capacity load. However, EAT is often associated with circulatory failure; therefore, we performed urgent radiofrequency (RF) catheter ablation of the EAT when he was 2 months and 11 days of age and weighed 3.1 kg. RF catheter ablation was performed under general anaesthesia (fentanyl and rocuronium) using an EnSite NavX system (St. Jude Medical, Minneapolis, MN, USA) (Supplementary material online, *Figure 1*). Electrode catheters and two temporary pacing leads attached during the prior surgery were positioned in the oesophagus, right atrium (RA), and RV. A 4 mm tip steerable catheter (Ablaze Fantasista, 5 F, Japan Lifeline, Tokyo, Japan) was used for ablation. Although antiarrhythmic drugs were discontinued 2 h before ablation, no EAT were induced during the electrophysiological study. Pace mapping suggested that the origin of the EAT could be at the RA–superior vena cava (SVC) junction. Circumferential ablation was performed ∼1 cm above the SVC–RA junction, except where diaphragmatic twitching occurred. The EAT disappeared after the RF catheter ablation. However, 2 weeks after surgery for phrenic nerve palsy, the EAT recurred, and the origin of the EAT was different from that of the EAT cauterized by RF catheter ablation in terms of P-wave morphology (*Figure 2C*). Antiarrhythmic drugs such as amiodarone, landiolol, and digoxin were ineffective, and nifekalant and flecainide were initiated. Ectopic atrial tachycardia is triggered by haemodynamic compromise, surgery, or other invasive procedures, resulting in shock, cardiopulmonary arrest, or stroke. We treated the cause of EAT, maximized medical therapy (Supplementary material online, *Table*), and performed catheter ablation. After a conference with a multidisciplinary department, the adverse effects of EAT and the advantages and disadvantages of AVN ablation and pacemaker implantation, such as heart failure, were discussed. We decided to perform urgent AVN ablation of the EAT when he was 3 months and 7 days old and weighed 3.1 kg. Radiofrequency catheter ablation was performed under general anaesthesia using a 4 mm tip steerable catheter (Ablaze Fantasista). We confirmed that temporary pacing leads of the RA and RV could be used before AVN ablation. The application was performed on the mid-septum of the tricuspid septum, where no HIS electrogram was recorded and a temporary AV block occurred during catheter manipulation (*Figure 2*). A complete AV block was achieved after one RF energy application in the temperature control mode (maximum 55°C, 40 W, and 60 s). The impedance dropped from 103 to 95 Ω during the application. Two additional applications were delivered to the same location. A permanent pacemaker was implanted after the ablation (Supplementary material online, *Figure 1*) and programmed to the DDD mode at rates of 130–170 ppm, 180-, mode switch to VVI 120 ppm, and a sensed/paced AV delay of 100/120 ms. After AVN ablation, the haemodynamics stabilized during the EAT. However, the patient died of mediastinitis at 4 months of age. On autopsy, myocardial necrosis and fibrous scarring were observed with Masson’s trichrome staining at the ablation sites, and most of the compact AVN were replaced. The width of the fibrotic lesion was 2.8 mm and the depth was 1.9 mm. No thrombosis was observed. The tricuspid valves were normal (*Figures 3* and *4*). The SVC lesion at the half-SVC ablation site exhibited fibrotic scarring.

## Discussion

**Figure 5:**
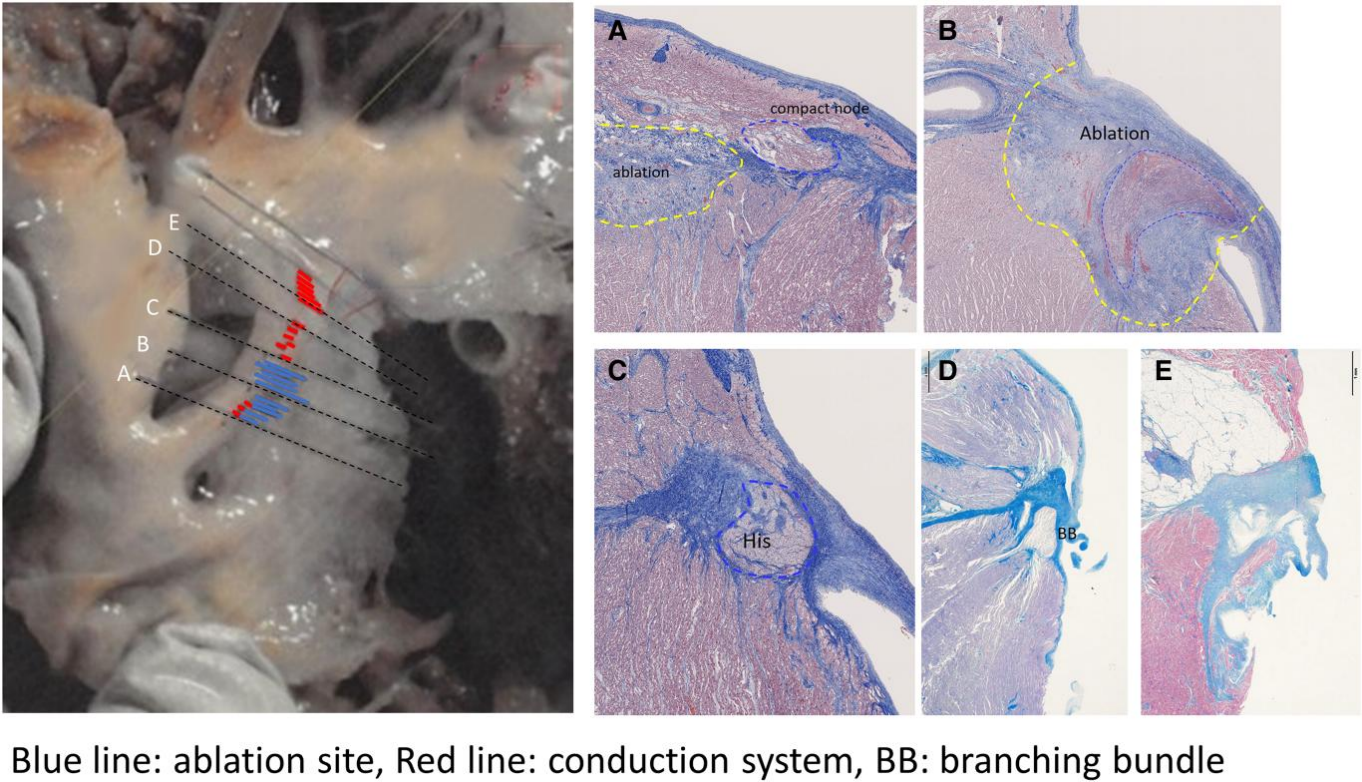
Detailed pathological findings of the atrioventricular conduction system. A view of the right ventricle shows Koch’s triangle with the boundaries of the coronary sinus orifice, tendon of Todaro, and the septal leaflet of the right atrioventricular valve. Left side of the figure: consecutive serial sections were made to evaluate the relationship between the conduction system and the ablation site. This series of histological sections, in a comparable orientation to the diagram, was taken through the His bundle and bundle branches to show the fibrous tissue surrounding the conduction tissue (Masson’s trichrome stain). The red lines indicate the conduction system, the blue lines indicate the ablation sites, and the black dotted lines indicate the representative planes of the histological sections (*A–E*). Post-cauterization was observed between points A and C, and the conduction system was disrupted between them. Compact atrioventricular node was confirmed in section A; however, section B exhibited necrosis, haemorrhage, and fibrous tissue around the atrioventricular conduction tissue due to ablation. Section C shows the atrioventricular conduction bundle surrounded by central fibrous tissue, and section D shows the bundle branches retaining their fibrous sheaths in the subendocardium of the sept.

Atrioventricular node ablation was successfully and effectively performed in a small infant with hypoplastic left-sided heart syndrome and a life-threatening EAT. The patient died from a bacterial infection 1 month after ablation. Histological findings revealed that the compact AV node was completely cauterized without damaging the tricuspid leaflets or coronary artery (*Figure 5*).

The causes of refractory atrial tachycardia in infants include perioperative complex heart disease, as in the present case, and atrial tachycardia from an arrhythmic substrate in the atrium, such as an atrial aneurysm or right atrial origin. In a study of 86 patients with hypoplastic left heart syndrome, arrhythmias occurred in a high proportion (57%), and the EAT was 13%. In most cases, arrhythmia resolves over time. Some of these cases can be treated by ablation or surgical resection. However, some arrhythmias cannot be controlled even with surgical resection. Surgical treatment to improve haemodynamics and the latter type of arrhythmia, which is difficult to control even after resection of the arrhythmic substrate, are considered indications for atrioventricular nodal ablation. The PACES/HRS expert consensus statement^1^ classifies AV ablation as class IIb when both medical therapy and ablation fail. Because paced patients with complex univentricular heart disease have a higher risk of mortality than those with native sinus rhythm and that the presumed ongoing atrial arrhythmias still pose an unmitigated thromboembolic risk, this approach should be used only as a last resort for symptomatic atrial tachycardia refractory to rate control. There have been two reports of AV node ablation after a Fontan operation.^5,6^ In this case, the patient had the fatal complication of atrial tachycardia. The origin of the atrial tachycardia after the first ablation was different from that of the first ablation in terms of the shape of the P-wave. Targeted arrhythmia was not induced during the first ablation, the origin was identified by pace mapping, and the SVC was isolated. Based on the above, it was uncertain whether a second ablation for atrial tachycardia would produce arrhythmia; therefore, we decided to perform AVN ablation.

An AV block was successfully created using RF energy of the mid-septum of the tricuspid annulus in a patient with hypoplastic left heart syndrome. Three RF applications with a 4 mm tip non-irrigated catheter were delivered: 55°C, 40 W, and 60 s, for a total energy of 6852 J. The ablation lesion had a width of 2 mm × 2.5 mm and a depth of 2 mm. The compact AVN was completely ablated without damaging the tricuspid leaflets or coronary artery (*Figure 5*). Although the site of the AV node in various complex congenital heart diseases is sometimes unclear, the AVN in the patient with hypoplastic left heart syndrome was similar to that in a normal heart. A previous study of hypoplastic left heart syndrome reported that the posterior atrioventricular junction exhibited a well-formed AVN just anterior to the mouth of the coronary sinus.^5,7^ We reported a case of successful AVN ablation in a 3-month-old infant with hypoplastic left heart syndrome and EAT. Following the AVN ablation and pacemaker implantation, haemodynamics stabilized. This clinical experience, which included the RF ablation and pathological findings, informed our decision-making regarding catheter ablation in infants.

## Supplementary Material

ytae493_Supplementary_Data

## Data Availability

Data on this case report can be made available on application.

## Lead author biography

Masayoshi Mori is a staff of paediatric cardiology at Osaka Women’s and Children’s Hospital, Osaka, Japan. He is interested not only in general paediatric cardiology but also in catheter ablation of children and congenital heart diseases.
