# Supplementary figures and images for: Atrioventricular node ablation and the pathological findings of a refractory ectopic atrial tachycardia in a small infant with hypoplastic left heart syndrome: a case report
Source: Eur Heart J Case Rep. 2024 Sep 10;8(9):ytae493. doi: 10.1093/ehjcr/ytae493 (PMC11413576; doi:10.1093/ehjcr/ytae493)

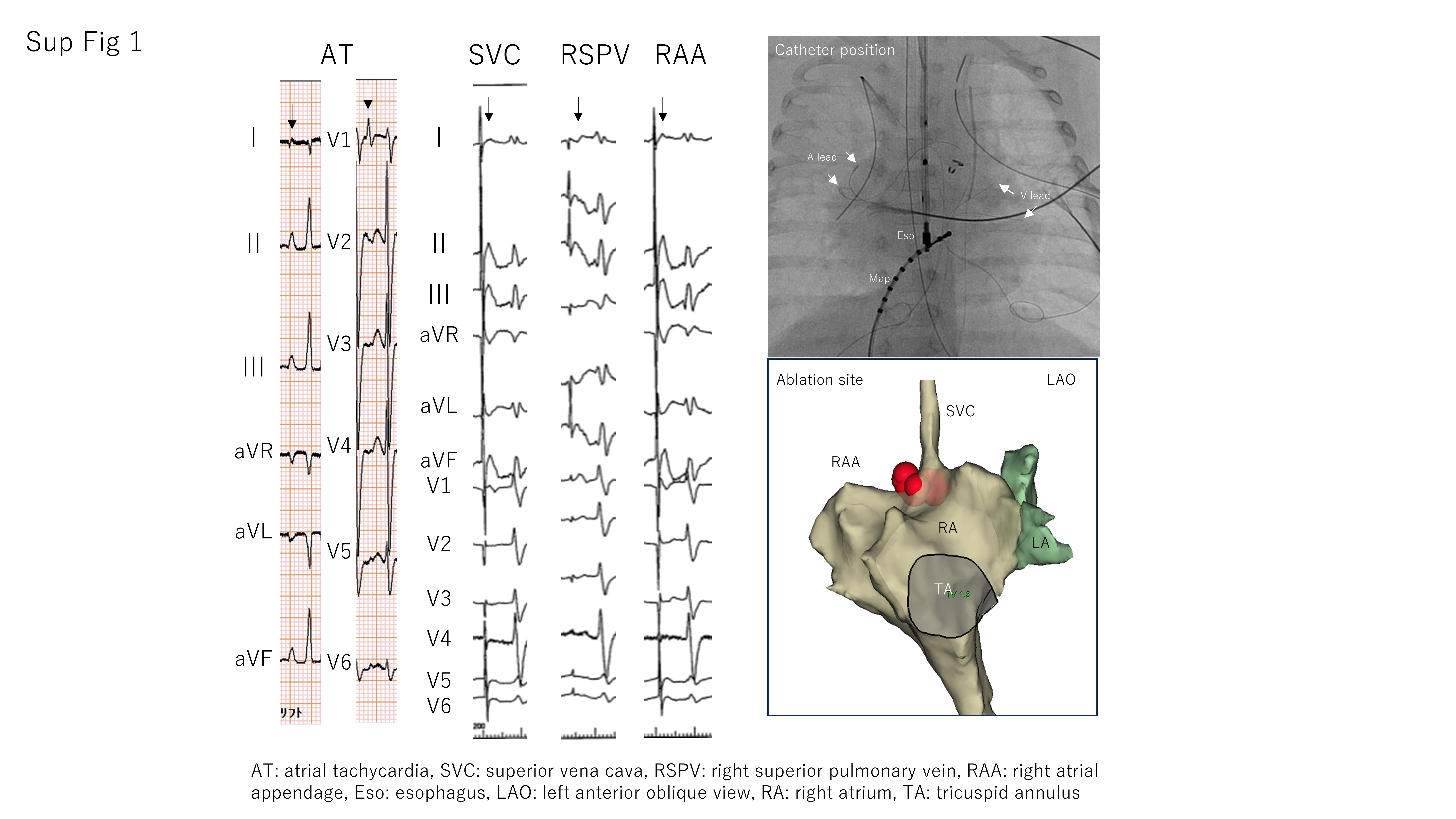

Supplement: ytae493_Supplementary_Data [file ytae493_supplementary_data.zip › Sup Fig 1.tif]

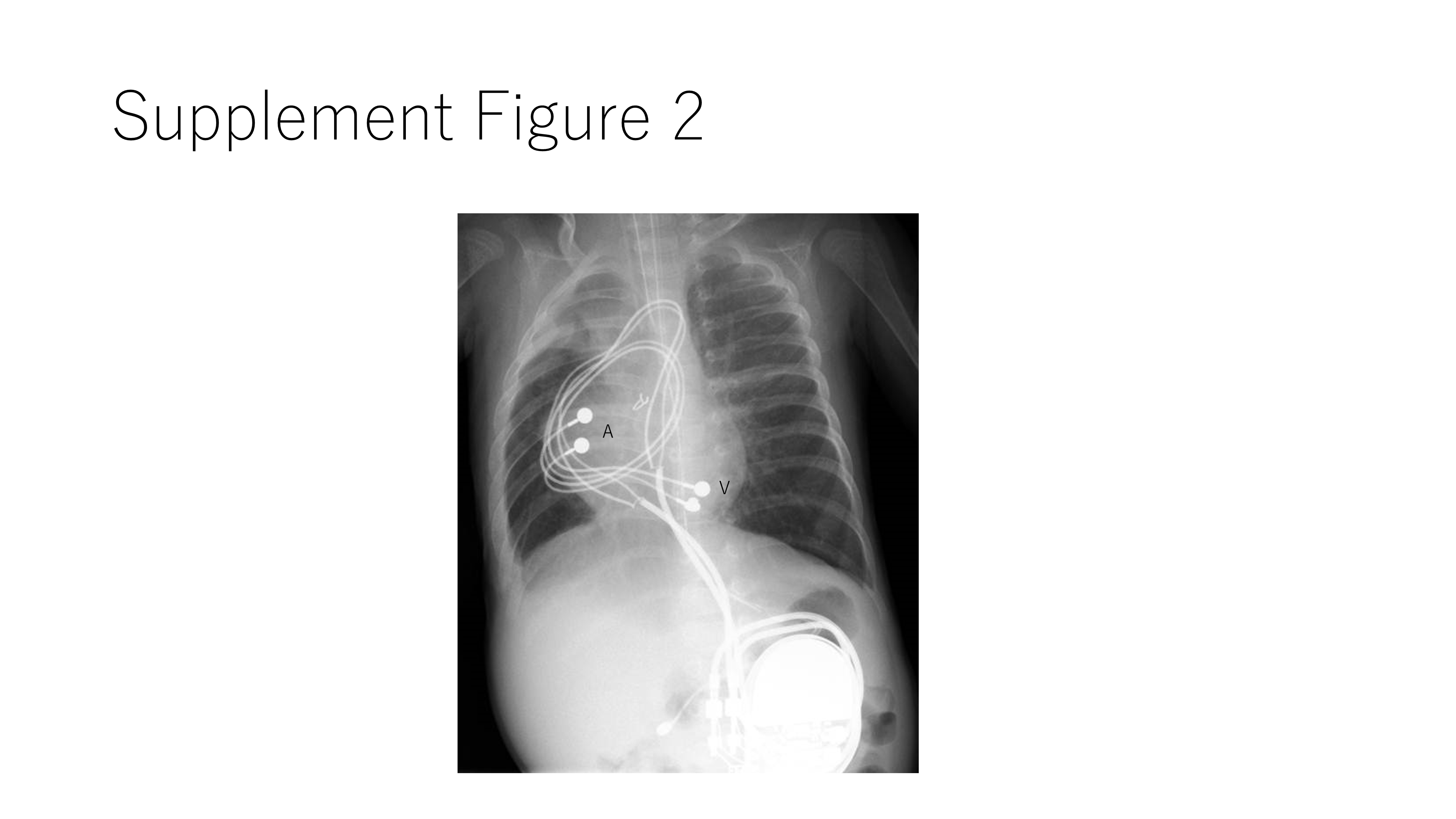

Supplement: ytae493_Supplementary_Data [file ytae493_supplementary_data.zip › Sup Fig 2.TIF]

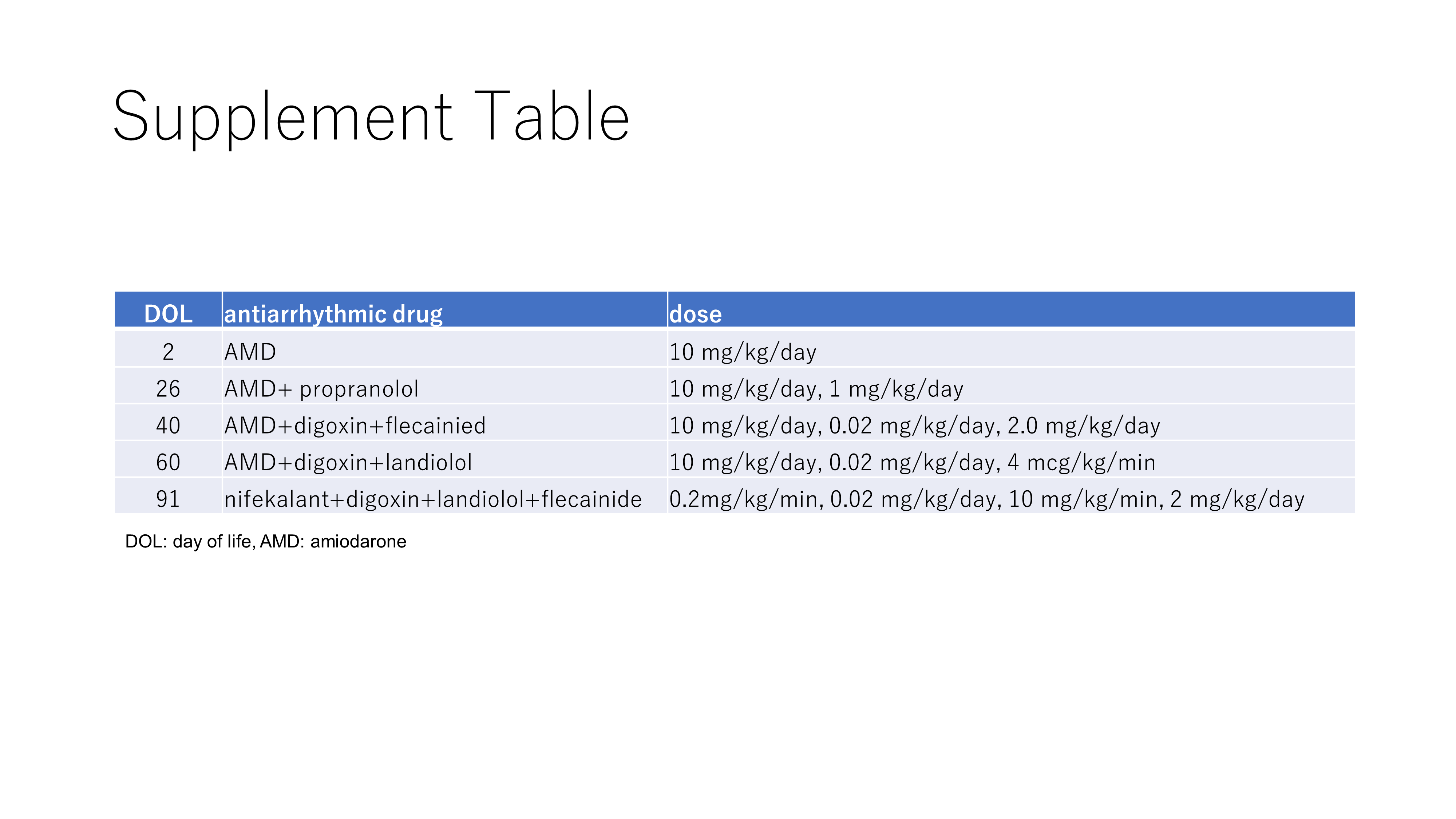

Supplement: ytae493_Supplementary_Data [file ytae493_supplementary_data.zip › Sup Table.TIF]
